# Review of Blood Pressure Control in Vulnerable Older Adults: The Role of Frailty and Sarcopenia

**DOI:** 10.3390/jvd4020018

**Published:** 2025-05-14

**Authors:** Kunaal S. Sarnaik, Saeid Mirzai

**Affiliations:** 1 Case Western Reserve University School of Medicine, Cleveland, OH 44106, USA; 2 Department of Cardiovascular Medicine, Wake Forest University School of Medicine, Medical Center Blvd, Winston-Salem, NC 27101, USA

**Keywords:** frailty, sarcopenia, older adults, hypertension management

## Abstract

The aging of the global population over recent decades has resulted in an increased prevalence of hypertension in older adults. Hypertension develops with increasing age primarily due to a disastrous feedback loop of increased arterial stiffness and maladaptive hemodynamics; this is compounded by age-related changes in physiology. The risk of adverse hypertension-related outcomes concurrently increases with age, and optimal blood pressure (BP) control in older adults thus becomes increasingly important each year. The results of several randomized clinical trials (RCTs) evaluating antihypertension strategies in older adults have concluded that the potential benefits of intensive BP management outweigh the risks of harm. However, the exclusion of frail, multimorbid, and institutionalized individuals limits the generalizability of such findings to the broader population of older patients with hypertension. Secondary analyses and external studies have continued to support intensive BP control strategies in older adults with frailty or sarcopenia. Therefore, based on available evidence, clinicians should continue practicing intensive BP control strategies in the older population, yet careful consideration of functional status, life expectancy, medication side effects, polypharmacy, and multimorbidity must take place to avoid unnecessary harm. Strategies must then be tailored to accommodate modifiers such as frailty and sarcopenia in older adults with hypertension. Knowledge gaps underscore the need for future studies evaluating BP management in older adults that incorporate greater proportions of multimorbid and institutionalized individuals with frailty, assess personalization of treatment, and identify subgroups in which optimal BP levels exist or the permissibility of higher BP levels is safer than BP reduction.

## 1. Introduction

### 1.1. Epidemiological Burden

With increasing life expectancy rates observed globally over recent decades, the world’s population continually ages [1]. The World Health Organization (WHO) recently projected the global population of people aged 60 years or older to double from ~1 billion in 2020 to 2.1 billion in 2050, and the proportion of those aged 80 years or older also to double from 12% in 2015 to 22% in 2050 [2]. Consequently, concurrent increases in the prevalence of chronic age-related diseases such as hypertension are also expected. In the United States (U.S.), a recent study estimated the prevalence of hypertension to increase from 51.2% in 2020 to 61.0% in 2050 [3]. Hypertension prevalence also increases with age; at least 72% of U.S. adults aged 65–74 have hypertension, increasing to at least 80% of those over 75 years old [4].

Hypertension is among the most common modifiable risk factors for cardiovascular disease, and from 2000 to 2018, the hypertension-related cardiovascular disease age-adjusted mortality rate has increased by 0.5% each year in the U.S. [5]. Several studies have found a stepwise increase in all-cause [6–8], cardiovascular-related [5,6,9], and renovascular-related mortality [10] with respect to increasing age of adults with hypertension. Morbidity measured by pooled outcomes such as major adverse cardiovascular event (MACE) rate or serious adverse event (SAE) rate has been found to increase with age in a similar stepwise fashion among adults with hypertension [11]. A meta-analysis of four randomized control trials (RCTs) [12–15] demonstrated 29%, 33%, and 37% reductions in MACE, cardiovascular mortality, and heart failure, respectively, with the implementation of intensive versus standard treatment of hypertension in adults over 65 years old [16]. However, a cross-sectional study utilizing National Health and Nutrition Examination Survey (NHANES) data found that approximately 33.2% of patients with treatment-eligible hypertension over 65 were left untreated; furthermore, a meager 56.7% of such patients that were treated were within goal blood pressure (BP) range.

### 1.2. The Frailty–Hypertension Interface

An explanation for the discrepancy between the repeatedly demonstrated benefits of antihypertensive treatment and the lack of guideline-based adherence in practice may be due to possible adverse effects of intensive BP therapy in older adults with frailty [17]. There is a myriad of evidence derived from prior RCTs such as HYVET [18], SPRINT [19], and STEP [20] supporting the lowering of BP in older individuals. However, common critiques of these trials involve their exclusion of patients with severe multimorbidity, disability, and institutionalization [21,22], factors bidirectionally related to frailty [23–26], limiting their generalizability. Moreover, observational studies of older patients with frailty have demonstrated increased mortality when BP is lowered beyond trial-derived targets [27–29]. Such findings suggest a U-shaped phenomenon of increased adverse events at either end of the BP spectrum in this population [30,31].

These findings, when combined with the aforementioned limitations of previous RCTs, are valid causes for concern when implementing intensive BP control in older adults with frailty. However, post hoc analyses of the SPRINT trial stratifying patients based on frailty [32] and sarcopenia [33] status continue supporting both the efficacy and safety of intensive BP control in this population. Furthermore, the observational studies may be inherently limited due to their retrospective nature and relatively less stringent BP measurement requirements. The low BP levels in these studies may also implicate alternative adverse underlying issues such as multimorbidity [34], malnutrition [35], or poor baseline health or functional status [36], instead of intensive BP control, as factors underlying the observed increases in mortality.

### 1.3. Scope and Methods

Given the complexity and intricacies inherent to managing BP and hypertension in the older population with frailty, it is important for healthcare providers to remain aware of relevant studies and up-to-date guidelines. In the present review, we briefly discuss the pathophysiological framework and clinical assessment of hypertension in the context of older age and frailty. We then summarize evidence amassed from relevant RCTs in this population and provide recommendations for treatment approaches and implementation strategies in the context of advanced age, frailty, and sarcopenia. Finally, we outline future directions and potential research gaps that must be addressed regarding intensive BP control in this vulnerable population. Regarding the literature search strategy, a narrative search of the MEDLINE database was conducted using the following keywords: (hypertension OR high blood pressure) AND (management) AND (elderly OR frailty OR sarcopenia). Studies were selected based on their relevance and potential to inform the research context.

## 2. Pathophysiological Framework

### 2.1. Vascular Aging

Increasing age remains a major non-modifiable risk factor for the development of hypertension [37]. As age advances, arteries progressively stiffen due to multiple factors primarily affecting the intimal and medial layers, including reduced and damaged elastin, increased collagen deposition, and calcification [38–40]. Such changes increase the thickness and stiffness of the vascular arterial wall. Furthermore, endothelial dysfunction increases with age. The subsequent increase in the production of vasoconstrictive mediators (e.g., endothelin-1) [41] with the concurrent decrease in that of vasodilatory substances (e.g., nitric oxide) [42] compounds arterial stiffness with advanced age. Increased arterial stiffness, in turn, leads to increased systolic BP secondary to reduced vascular compliance [43]. This causes further progression of the elastin-, collagen-, calcium-, and endothelium-related changes, creating a disastrous bidirectional feedback loop of increasing arterial stiffness and BP over time [44].

Together, these factors and the associated decrease in arterial elasticity, especially in large arteries, also lead to impaired baroreceptor sensitivity [45]. Since the baroreceptor reflex responds to varying levels of arterial strain with autonomic- and hormone-mediated changes in vasomotor activity [46], blunted baroreceptor sensitivity due to arterial stiffness results in high BP variability with increasing age [47,48]. BP variability has been described previously as an independent predictor of end-organ damage and several adverse outcomes ranging from MACEs to all-cause mortality [48]. BP variability has also been demonstrated to play a role in the aforementioned feedback loop between arterial stiffness and BP [49]. Particularly, BP variability in the aging population is notable with positional changes, leading to various phenomena including orthostatic hypotension [50] and supine hypertension [51].

### 2.2. Hemodynamic Changes

The aforementioned decrease in vascular compliance secondary to increased arterial stiffness with advanced age not only increases systolic BP but may also lead to slight decreases in diastolic BP [52]. The pulse pressure consequently widens with increasing age. Thus, in contrast to younger adults, where diastolic BP is a more important prognostic factor for hypertension [53], increasing systolic BP becomes the main driver of hypertension in older adults, and many have isolated systolic hypertension [54]. Furthermore, isolated systolic hypertension has been repeatedly demonstrated to be independently associated with cerebrovascular, cardiovascular, and all-cause mortality in the older population [55–57].

Pulse pressure has also been shown to be an equally important prognostic factor in older adults with hypertension, with increasing pulse pressure being a strong predictor of both cardiovascular and all-cause mortality [58]. In older adults with hypertension, systolic BP and pulse pressure are thus more important determinants of cardiovascular disease and mortality risk relative to diastolic BP [59]. Isolated diastolic hypotension in the older population has also been demonstrated to increase the risk of adverse outcomes such as incident heart failure [60], cardiovascular mortality [61], and all-cause mortality [61,62].

### 2.3. Age-Related Physiological Changes

The age-related changes in vascular structure and function, as well as hemodynamics, are further compounded by changes in pharmacokinetics and pharmacodynamics associated with increasing age. In the older population, reduced absorption, reduced renal and hepatic clearance of water- and lipid-soluble pharmacotherapies, and increased volume of distribution of lipid-soluble pharmacotherapies, is observed [63]. Such changes have been demonstrated to complicate the treatment of hypertension in older adults [64]. Moreover, the older population’s decreased absorption of nutrients may also result in phenomena such as postprandial hypotension due to the aforementioned impaired baroreceptor response [65]. Postprandial hypotension further complicates antihypertensive treatment and is an independent predictor of cardiovascular disease and all-cause mortality in this population [66].

There are also notable changes in the renin–angiotensin–aldosterone system (RAAS) [67] and the beta-adrenergic response [68] with increasing age. Specifically, circulating RAAS is suggested to be suppressed [67], and beta-adrenergic responsiveness decreases in a process known as “beta-adrenergic desensitization” [68]. Such changes, in combination with multimorbidity, polypharmacy, and frailty, affect the pharmacodynamics of various antihypertensive treatments in older adults [64]. Consequently, expert consensus recommends initiating low dosages with stepwise increases over time when prescribing antihypertensives in the older population with frailty [69], as described in more detail later.

## 3. Clinical Assessment

### 3.1. BP Classification

According to the 2017 American College of Cardiology (ACC)/American Heart Association (AHA) hypertension guidelines [70], BP is categorized as normal (<120/80 mmHg), elevated (120–129/<80 mmHg), stage 1 hypertension (130–139/80–89 mmHg), and stage 2 hypertension (≥140/90 mmHg) based on ≥2 careful readings obtained on ≥2 separate occasions (Table 1). Office BP readings outlined by the 2024 update of the 2018 European Society of Cardiology (ESC)/European Society of Hypertension (ESH) hypertension guidelines [71] are similarly categorized as non-elevated (<120/70 mmHg), elevated (120–139/70–89 mmHg), and hypertension (≥140/90 mmHg) (Table 1).

Treatment thresholds outlined by both sets of guidelines vary based on the degree of cardiovascular disease, and the presence, absence, or risk of cardiovascular disease is an important qualifier to BP classification. According to the 2017 ACC/AHA guidelines [70], for adults meeting criteria for the elevated or stage 1 hypertension categories without history of cardiovascular disease and an estimated 10-year atherosclerotic cardiovascular disease (ASCVD) risk of less than 10%, non-pharmacologic therapy (e.g., weight loss, heart-healthy diet, sodium restriction, potassium supplementation, increased physical activity, and alcohol restriction) is the mainstay of antihypertensive management. On the other hand, if there is presence of cardiovascular disease or the estimated 10-year ASCVD risk is greater than or equal to 10% in the elevated or stage 1 hypertension categories, pharmacologic therapy is additionally recommended. In stage 2 hypertension, drug therapy is recommended for all patients, regardless of cardiovascular disease history or 10-year ASCVD risk [70]. Similar antihypertensive treatment recommendations are outlined in the ESC/ESH guidelines [71].

### 3.2. Risk Assessment

**Figure 1.**
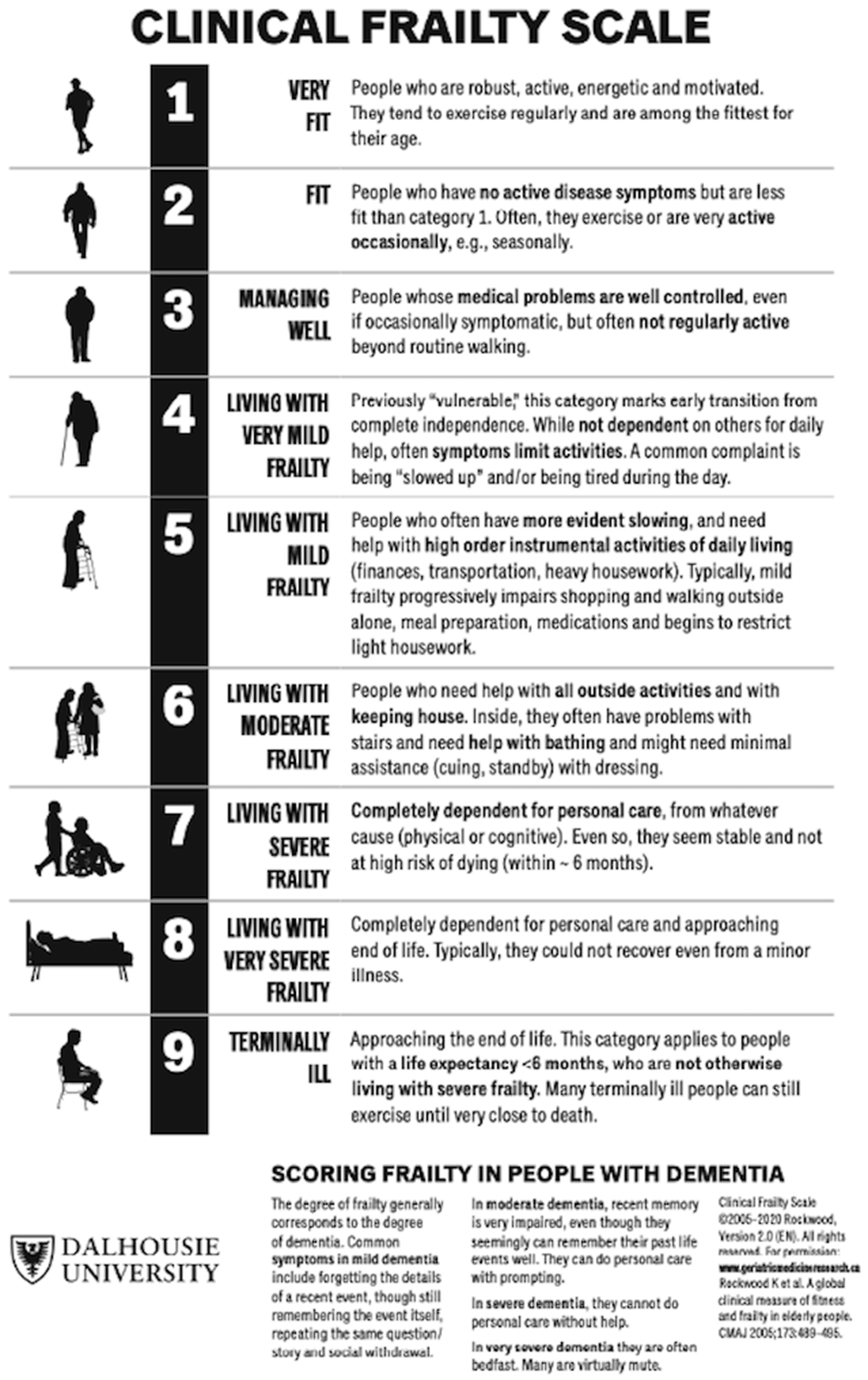
2020 Clinical Frailty Scale (CFS) tool developed from the Canadian Study of Health and Aging (CSHA) to stratify older patients based on degree of clinically relevant frailty. From Rockwood and Theou [74], with permission; © 2005–2020 Rockwood Version 2.0 (EN).

Frailty is an age-related multidimensional geriatric syndrome marked by decreased physiologic reserve and function and increased vulnerability to stressors [72]; the presence and degree of frailty should be accounted for in older adult patients with hypertension. The Clinical Frailty Scale (CFS) developed from the Canadian Study of Health and Aging [73] is a valuable and efficient tool for such identification and stratification. Recently updated in 2020 [74] and validated for various applications thereafter [75–78], the nine-point CFS guides clinicians into stratifying older patients into the following categories: (1) very fit, (2) well, (3) managing well, (4) living with very mild frailty, (5) living with mild frailty, (6) living with moderate frailty, (7) living with severe frailty, (8) living with very severe frailty, and (9) terminally ill [72] (Figure 1). The CFS has been demonstrated to be an important prognostic factor in older adults with hypertension [79], as well as in other cardiovascular applications such as heart failure [80] and coronary artery or valvular surgeries and procedures [81].

**Figure 2.**
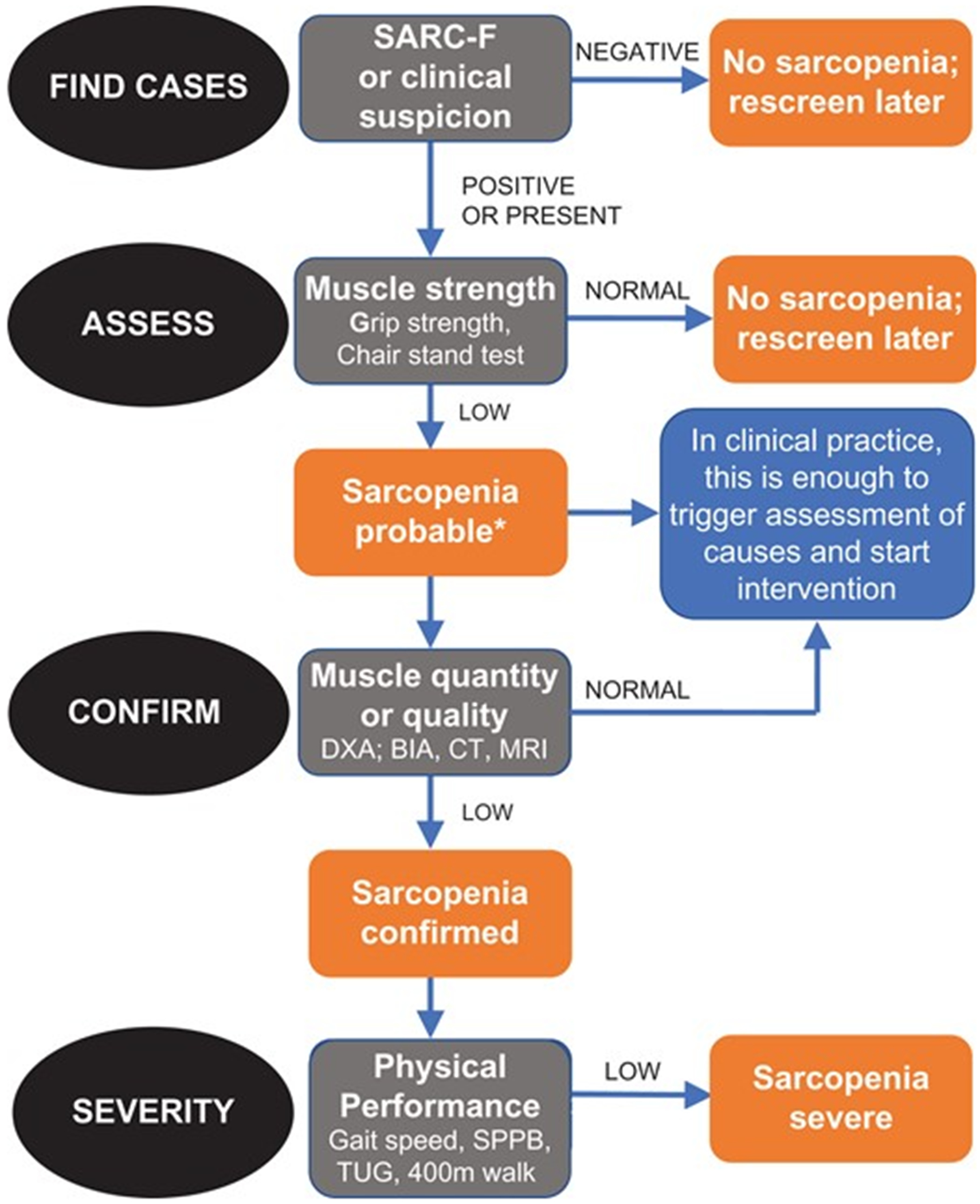
Algorithm for diagnosing and stratifying sarcopenia in clinical practice outlined by the European Working Group on Sarcopenia in Older People (EWGSOP). From Cruz-Jentoft et al. [87], with permission; © 2018 Oxford University Press.

The same risk assessment should also be performed in older adults for sarcopenia, the age-related degradation of muscle strength, mass, and performance [82]. Here, it is important to contrast frailty with sarcopenia. Frailty is the gradual multisystem loss of physiologic reserve, and its pathogenesis is complex, multidimensional, and typically identified late in its natural history, even with prognostic tools such as the CFS [83]. Sarcopenia, on the other hand, is specific to muscle and estimated to be approximately twice as common as frailty [84]. Unlike frailty, which is difficult to diagnose due to late functional decline and heavy reliance on clinical judgement [85], sarcopenia has relatively more concrete diagnostic criteria established by consensus definitions such as those from the European Working Group on Sarcopenia in Older People (EWGSOP) [86,87]. The most recent EWGSOP2 [87] guidelines define sarcopenia as loss of muscle strength and mass (e.g., quantity or quality). The qualifier of severe sarcopenia is added when there is also evidence of decreased muscle performance [87]. The EWGSOP2 additionally outlined relatively simple and objective techniques for evaluating muscle strength (grip strength and chair stand test), muscle quantity or quality (appendicular skeletal muscle mass {ASMM} by dual-energy X-ray absorptiometry {DXA}, whole-body skeletal muscle mass {SMM} or ASMM by bioelectrical impedance analysis {BIA}, mid-thigh muscle cross-sectional area by computer tomography {CT} or magnetic resonance imaging {MRI}, lumbar muscle cross-sectional area by CT or MRI, and muscle quality by mid-thigh or total body muscle quality by muscle biopsy, CT, MRI, or magnetic resonance spectroscopy {MRS}), and muscle performance (gait speed) [87] (Figure 2).

The identification of sarcopenia is consequently relatively more straightforward than that of frailty, and sarcopenia gives clinicians an important qualifier of aging that may be more concrete to measure and track earlier in its natural history relative to frailty through the use of simpler and widely available, easily performable tests. Sarcopenia also commonly leads to frailty [84] or modulations within the degree of frailty [88]. Moreover, sarcopenia has been identified as a risk factor for the development and faster progression of cardiovascular diseases [89,90]; loss of muscle predisposes patients to insulin resistance, chronic inflammation, physical inactivity, and malnutrition [91]. Thus, early identification and treatment of sarcopenia is important when addressing the management of hypertension in the older population with frailty, and even more so considering recent studies have demonstrated significant associations between sarcopenia and hypertension in older adults [92,93].

In addition to frailty and sarcopenia, risk assessment of hypertension in the older population with frailty must consider the role of multimorbidity, polypharmacy, mental health, ambulation status, and life expectancy. Multimorbidity has been demonstrated to play an important role in the control rate of hypertension in this population [94]. Polypharmacy is a common finding in older adult patients with hypertension that increases the rate of drug–drug or food–drug interactions [94]. Polypharmacy has also been found to be an independent predictor of hospitalization and all-cause mortality in older individuals [95]. Furthermore, mental health has been described to have a bidirectional relationship with hypertension, with the coexistence of mental health problems and hypertension resulting in decreased quality of life and treatment adherence, as well as higher mortality [96]. Ambulation status is also an important factor in risk assessment, as hypertension in the older population with frailty leads to increased fall risk [97]. Finally, the role of life expectancy in risk assessment of any disorder such as hypertension in older adults is important, as intensive treatment recommendations in this population may confer more harm than benefit when limited life expectancy is considered [98].

## 4. Major Trial Evidence

### 4.1. HYVET Trial

Previous RCTs have evaluated the role of intensive BP management in the older population. The first major trial of interest was HYVET (2001 to 2007), a multinational RCT of 3845 participants over 80 in Europe, China, Australasia, and Tunisia with preexisting systolic BPs of >160 mmHg [18]. The treatment arm of the investigation received indapamide initially, with perindopril added if needed. The treatment arm had a target BP of <150/80 mmHg, and outcomes of this group were compared to that of a placebo-controlled arm over a median follow-up of ~2 years [18]. The results of HYVET demonstrated active treatment to reduce the rate of stroke-related mortality by 39%, all-cause mortality by 21%, and cardiovascular mortality by 23% relative to the control arm [18].

HYVET was among the first large-scale studies to concretely demonstrate the beneficial impact of antihypertensive therapy in older adults [99]. Specifically, HYVET found that even in patients above 80 with hypertension, initiating antihypertensive therapy is not too late to prolong survival. However, critiques of HYVET commonly question the generalizability of the participant cohort. In HYVET, good physical and mental health was an inclusion criterion, and the RCT excluded participants with dementia and those institutionalized in nursing homes [100]. The HYVET trial was also potentially marked by limited adverse event reporting along with lack of relevant details in the publication; only five undefined adverse events were attributed to study medications by the investigators [101]. Moreover, orthostatic hypotension and hyponatremia, known side effects of thiazide diuretics such as indapamide [102,103] as well as common general developments in the older population [104,105], were not reported as adverse events in the HYVET trial [101].

### 4.2. SPRINT Trial

The SPRINT RCT (2010 to 2015) compared intensive (<120 mmHg) versus standard (<140 mmHg) antihypertensive therapy achieved using various antihypertensive medications in 9361 non-diabetic participants in the U.S. aged 50 years or older with pre-existing systolic BP readings of 130 to 180 mmHg [19]. After a median follow-up of ~3 years, the SPRINT investigation was terminated due to the significant benefits established in the intensive BP control arm relative to those in the standard BP control arm [19]. Specifically, the results of SPRINT demonstrated a 25% reduction in the rate of developing the primary composite outcome of myocardial infarction, other acute coronary syndromes, stroke, heart failure, or cardiovascular mortality with intensive relative to standard BP control [19]. The investigation also found a 27% reduction in the rate of all-cause mortality with intensive relative to standard BP control [19]. These results were even more pronounced in the subgroup analysis conducted among 2636 SPRINT participants aged 75 or older, with 34% and 33% reductions in the observed rates of the primary outcome and all-cause mortality, respectively [13]. Furthermore, cognitive assessments administered to participants in the SPRINT trial demonstrated significant reductions in the risk of mild cognitive impairment and the combined rate of cognitive impairment and dementia [106]. An extended follow-up analysis conducted through the end of 2023 on as many as 8563 participants is hypothesized to further bolster these findings [107,108]. Finally, a sub-study of MRI findings in 670 SPRINT subjects demonstrated smaller increases in cerebral white matter lesions among participants receiving intensive treatment than those receiving standard treatment [109].

A limitation of the SPRINT intervention is the increased risk of serious adverse events of hypotension, syncope, electrolyte abnormalities, and acute kidney injury observed in the intensive treatment arm relative to the standard treatment arm in the main trial [19]. Importantly, however, the overall rates of serious adverse events were not different between the intensive and standard treatment arms in the subgroup analysis of participants aged 75 and older [13]. In addition, there were no differences in the isolated rates of hypotension, syncope, electrolyte abnormalities, acute kidney injury, and injurious falls in this subgroup analysis of older adult patients [13]. Yet, criticism of SPRINT was similar to that of HYVET; the exclusion criteria of participants with pre-existing dementia and those who resided in assisted-living facilities or nursing homes may have limited the generalizability of the older adult SPRINT participants to the overall population of older adults with hypertension who are commonly frail, multimorbid, and disabled [110]. Furthermore, the SPRINT RCT also excluded participants with a history of stroke or diabetes, as well as those with limited life expectancy [19]. A cross-sectional, population-based investigation, however, refuted such criticism, demonstrating that a substantial percentage of adults in the U.S. met the eligibility criteria for SPRINT [111]. Regardless, the generalizability of SPRINT to older individuals with frailty or sarcopenia remains a subject of debate [112].

Post hoc analyses of SPRINT have attempted to address this question. In 2023, Wang et al. [32] conducted such an analysis of 2560 SPRINT participants identified to have frailty based on the 36-item frailty index constructed by the SPRINT study group [113]. The investigation found that though frailty portends a significantly higher risk of developing the primary outcome in both treatment arms, there was no difference in intensive treatment effects on the primary outcome with respect to frailty status [32]. Furthermore, the post hoc study did not find a difference in the risk of developing serious adverse events with respect to the interaction of frailty and intensive BP treatment [32]. Thus, the Wang et al. investigation [32] provides evidence that participants with frailty in SPRINT benefit similarly to participants without frailty with respect to intensive BP control without an increased risk of serious adverse events.

Additionally, a recent post hoc analysis conducted by Mirzai et al. in 2024 among SPRINT participants evaluated sarcopenia as a potential qualifier of the trial’s conclusions [33]. Using the serum-based sarcopenia index and gait speed data in the subgroup of 2571 SPRINT participants aged 75 or older, the secondary analysis demonstrated that sarcopenia, similar to frailty in the Wang et al. investigation [32], was an independent risk factor for cardiovascular events and all-cause mortality in SPRINT participants [33]. However, in SPRINT participants found to have sarcopenia, Mirzai et al. found intensive BP control to nearly halve the risk of cardiovascular events without increasing the risk of serious adverse events relative to participants receiving standard BP control [33]. Furthermore, the investigation reported decreased all-cause mortality of participants with sarcopenia receiving intensive BP control relative to those receiving standard BP control when stratified by the presence of chronic kidney disease [33]. Together, the results of the post hoc Wang et al. [32] and Mirzai et al. [33] investigations provide evidence supporting intensive BP control recommendations in older participants with hypertension and frailty or sarcopenia.

### 4.3. STEP Trial

The most recent major RCT evaluating intensive BP control in the older population with hypertension was the STEP trial (2017–2020) [20], which was conducted in 60- to 80-year-old Chinese participants with hypertension and included diabetics. The STEP trial compared intensive treatment (110–130 mmHg systolic BP target) to standard treatment (130–150 mmHg systolic BP target) in 8511 Chinese adults over a median follow-up period of ~3.5 years [20]. The primary outcome of STEP was a composite of stroke, acute coronary syndrome, acute decompensated heart failure, coronary revascularization, atrial fibrillation, or cardiovascular mortality [20]. The results of the STEP trial demonstrated a 26% risk reduction of the primary outcome among participants in the intensive treatment arm relative to those in the standard treatment arm [20].

The findings of the STEP trial [20] generally agree with those of the SPRINT trial [19] with respect to the benefits of intensive antihypertensive therapy in the older population outweighing potential harms. Though the STEP trial did not find differences in the development of the primary outcome in the subgroup analysis of participants aged 70 to 80 years old, the study did not include any participants over 80 [20]. Furthermore, safety and adverse renal outcome rates did not differ between the intensive and standard treatment arms in the STEP trial [20]. However, similar to SPRINT [19], the risk of hypotension in the intensive treatment arm of STEP was greater than that of the standard treatment arm [20]. Also similar to both HYVET [18] and SPRINT [19], the STEP trial [20] excluded participants with severe cognitive impairment or mental disorders, as well as those with a history of stroke. Therefore, concern remains regarding the generalizability of the STEP trial to older individuals with frailty or sarcopenia. Secondary analyses may be necessary to further describe any qualifying effects of these age-related conditions on the outcomes of STEP.

## 5. Treatment Approaches

### 5.1. Treatment Stratification by Functional Status

**Figure 3.**
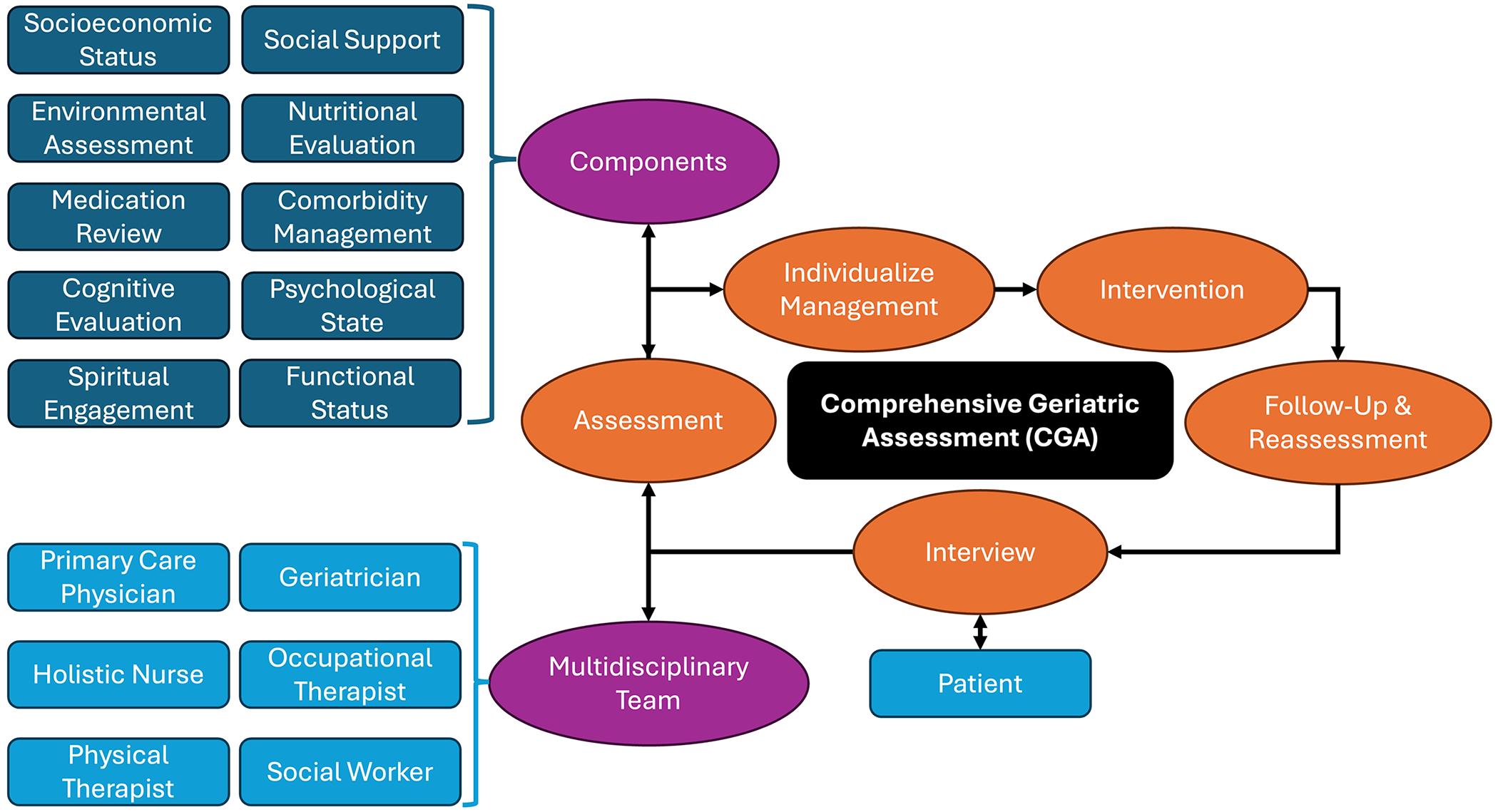
Overview of the comprehensive geriatric assessment (CGA). A multidisciplinary team interviews and independently assesses various components of each patient’s life and health prior to individualizing management and periodically following up and reassessing to properly tailor management.

In both sets of hypertension guidelines outlined by the ACC/AHA [70] and ESC/ESH [71] mentioned earlier in this review, important qualifications for treatment recommendations for the older population with frailty are given, yet they remain relatively unclear regarding identifying frailty status (Table 1). There thus exists a need for a comprehensive framework outlining best practices for the management of hypertension in the older population that additionally account for frailty or sarcopenia. The comprehensive geriatric assessment (CGA) is one such strategy, in which a multi-dimensional multi-disciplinary diagnostic process is pursued with the involvement of a physician, a geriatrician, a holistic nurse, an occupational therapist, a physiotherapist, and a social worker for each older patient with frailty [114] (Figure 3). The CGA has repeatedly demonstrated morbidity and mortality benefits among this population [115,116], and the thoroughness of the CGA may be valuable in determining important personalization factors when prescribing antihypertensives to older adults. However, the CGA has numerous limitations, primarily concerning the high degree of complexity in the diagnostic process. Firstly, several partnerships between the aforementioned disciplines involved in the CGA must be formed, and this is challenging to sustain over time [117]. Similarly, the CGA is a time-consuming and relatively expensive process that may be difficult to gain access to among patients who have suboptimal access to care, treatment adherence, or value for preventative services [117]. Finally, there is a lack of standardization in the CGA across domains, compounded by differences in its delivery and performance across multiple settings [118].

The CGA may consequently not be readily implementable and effective for older patients with hypertension, and more rapid, widely available, and easily performable assessments focused on functional status should instead be performed. One such framework was recently proposed in the literature by Benetos et al. [119] in 2019 by utilizing the previously discussed CFS. In their decision algorithm, Benetos et al. [119] describe further stratifying the nine-point CFS scale into three patient profiles based on functional status: (group 1) preserved function (CFS score of 1–3), (group 2) loss of function/preserved activities of daily living (ADLs) (CFS score of 4–5), and (group 3) loss of function and altered ADLs (CFS score of 6–9) (Table 2). For group 1 patients, Benetos et al. [119] recommend an antihypertensive approach similar to younger adults with adherence to the established guidelines [70,71], yet starting with monotherapy and titrating antihypertensive medications slowly. For group 3 patients, the authors suggest a more cautious approach, starting with monotherapy and keeping conservative goals of systolic BPs < 150 mmHg [119]. They also recommend avoiding the use of >3 antihypertensive medications in group 3 patients, carefully monitoring for the development of systolic BPs < 130 mmHg or orthostatic hypotension and rapidly reducing dosages if these findings were to manifest [119]. Finally, for group 2 patients, Benetos et al. [119] recommend performing a CGA and, based on the degree of altered functional status found, adopting the recommendations of either group 1 or group 3. Thus, the decision workflow proposed by Benetos et al. [119] is quick, efficient, and performable by virtually all physicians. Furthermore, it only pursues the time-consuming and expensive CGA if the degree of functional status and ADL loss is equivocal.

### 5.2. Pharmacological Management

For pharmacological management of hypertension in older adults, first-line agents include thiazide diuretics (TZDs) or calcium-channel blockers (CCBs) [120]. If a third antihypertensive agent is required, angiotensin-converting enzyme inhibitors (ACEis) or angiotensin receptor blockers (ARBs) may be added, followed by mineralocorticoid receptor antagonists (MRAs) [120]. Beta-blockers (BBs) are not generally considered first-line agents in patients aged 60 or older due to demonstrated inferiority compared to the other agents outlined above [121,122]. BBs are instead reserved for specific indications in older adults with hypertension, such as coexisting heart failure or ischemic heart disease [123]. Special considerations also apply to older patients with coexisting hypertension and chronic kidney disease (CKD) stage 3 or higher, where ACEis (or ARBs, if intolerant) are preferred to slow the progression of CKD [120]. Similarly, RAAS inhibitors have been recently demonstrated to potentially slow the progression of sarcopenia and may be emerging as a preferred treatment in older patients with hypertension that have evidence of decreased muscle strength, mass, or performance [124].

Clinicians should avoid the use of non-selective peripheral alpha-1 adrenergic antagonists and central alpha-agonists for the treatment of hypertension in older adults [125]. Both agents inflict a high risk of orthostatic hypotension on this population, which may lead to falls and increased morbidity and mortality [125]. Furthermore, central alpha-agonists have a high risk of adverse central nervous system (CNS) effects and bradycardia [125]. Certain combinations of drugs should also be avoided. For instance, the negative chronotropic effects of non-dihydropyridine CCBs and BBs may result in atrioventricular block or bradycardia [126]. Additionally, dual RAAS blockade (i.e., ACEi with ARB) regimens are generally not recommended [126]; the ONTARGET trial demonstrated an increased frequency of adverse events without an increase in benefit in the dual RAAS blockade treatment arm [127].

An important consideration in this discussion is that most older patients will require two or three antihypertensive medications to reach target BP ranges [128]. However, this may conflict with recommendations for the cautious, slow titration (“start low, go slow” method) of antihypertensives outlined by Benetos et al. [119] and other experts [64]. While the slow titration of antihypertensives may intuitively decrease the risk of avoidable adverse drug reactions or drug–drug interactions, it may also increase the time spent continually re-evaluating the efficacy of each stage of titration. This, in turn, may delay important risk reduction of escalating antihypertensive treatment. Given that first-dose hypotension and the highest frequency of adverse drug reactions occur in the first 1 to 2 weeks after starting treatment [64,129], it may be beneficial to adopt more aggressive patterns of titration for patients in the preserved function group (i.e., higher degrees of functional status) after this period. Nevertheless, establishing specific thresholds for monotherapy or combination therapy in the older population is an important area of future research [64].

Finally, an important consideration of pharmacologic intervention for hypertension management in the older population involves the “domino” effect commonly observed in individuals with frailty [130] This is a phenomenon wherein the multiple, interrelated systems and factors bidirectionally contributing to frailty make it such that even a small adjustment to therapy may overcome a precarious threshold and lead to rapid health destabilization [130] Thus, in older patients with frailty that have higher degrees of functional impairment, careful consultation of any antihypertensive regimen changes from experienced geriatricians and pharmacists may be paramount to avoid such “domino” effects.

### 5.3. Non-Pharmacological Interventions

Non-pharmacological interventions should be pursued in all individuals with elevated BP or hypertension [70,71], though special considerations should be applied in older adults. The Dietary Approaches to Stop Hypertension (DASH) diet [131] has been found to independently decrease systolic BP by 8–14 mmHg [128]. Additionally, sodium intake should be reduced to ≤2.4 g of sodium per day with demonstrated benefits of 2–8 mmHg reductions in systolic BPs [128]. Potassium supplementation through increased consumption of foods such as fruits and vegetables has also demonstrated beneficial effects on systolic BP reduction in patients with hypertension [132]; the AHA recommends potassium intake of 3000 mg/day for men and 2300 mg/day for women [133]. As always, physicians should also counsel older patients with hypertension on moderating alcohol consumption. Upper limits of two drinks per day for men and one drink per day for women have been shown to reduce systolic BP by 2–4 mmHg [128]. Similarly, increasing physical activity is an important non-pharmacological intervention in the older population. Physical activity recommendations include ~30 min of aerobic activity per day, most days per week, which can reduce systolic BP by 4–9 mmHg [128].

Many of these non-pharmacological interventions may lead to weight reduction. Weight reduction has also been independently demonstrated to have beneficial effects on BP reduction, with 10 kg of weight loss corresponding to 5–20 mmHg reductions in systolic BP [128]. However, skeletal muscle may also be lost with fat, leading to worsened body composition and increased cardiovascular disease risk through previously outlined pathways implicated in sarcopenia [134]. Resistance training may help combat the decrease in muscle mass associated with weight reduction in older adults and should be incorporated into aerobic exercise routines [135,136]. Importantly, due to the increased use of glucagon-like peptide-1 receptor agonists (GLP1RAs) for applications such as weight loss observed over recent years [137], providers must be keenly aware regarding the potential for substantial muscle loss associated with GLP1RA use [138,139]. Thus, as the proportion of older adults using GLP1RAs increases [140], future studies evaluating the risk of muscle loss and potential interventions such as resistance training with such medications are necessary to better inform decision-making regarding weight reduction with GLP1RAs in the older adult population in the context of reducing BP.

## 6. Implementation Strategies

### 6.1. Practical Management

Older adult patients should present to the clinic monthly as medications are initiated and titrated to achieve BP goals [141]. A longer interval of 3–6 months between clinic visits may be pursued thereafter without a demonstrated increase in risk of adverse cardiac events or all-cause mortality in patients with hypertension [142]. Home BP monitoring should also be performed at least 3–4 days per week with four measurements per day, two each morning and two each evening taken 1–2 min apart [143]. Furthermore, using fixed-dose combinations and 90-day prescription refills is encouraged when possible, as these strategies have been shown to increase treatment adherence [144,145].

For patients with coronary artery disease, careful and regular monitoring of diastolic BP is important, as both diastolic hypotension (<60 mmHg) [146] and diastolic hypertension (>90 mmHg) [147] are risk factors for cardiovascular disease events. Renal function should also be monitored regularly, especially early on in treatment, ~2–4 weeks after initiating RAAS inhibitors or diuretics [70]. Furthermore, if BP goals are unmet, evaluation and assessment of social barriers may be indicated. Importantly, the management of hypertension in older individuals with frailty requires a team-based approach with an emphasis on shared decision-making. Special consideration must be taken to align treatment with patient preferences and values, as well as the input of family members and caregivers.

### 6.2. Special Considerations

Orthostatic hypotension, as discussed previously, is common in older adults, even among those who are hypertensive [105]. Providers should always assess for symptoms of orthostatic hypotension, such as light-headedness or syncope, in this population, especially when prescribing antihypertensive drugs. The presence of orthostatic hypotension requires specific monitoring and lifestyle adaptations. Specifically, measures include physical counter-maneuvers, head-up sleeping, salt/fluid repletion, educating patients on avoiding triggers, having men sit to urinate, and implementing regular monitoring strategies [105]. Furthermore, the presence of orthostatic hypotension should always prompt a thorough medication review for reversible causes, and medication changes should be implemented if necessary.

Malnutrition has also been described as an independent risk factor for all-cause and cardiovascular mortality in the older population with hypertension [148]. More generally, nutrition status can affect BP management and may require medication adjustment. Therefore, it is important for providers to be aware of nutritional status changes in older patients with hypertension and properly counsel this population on the importance of maintaining a stable, healthy diet. Weight stability is similarly associated with lower mortality risk in this population [149] and thus should also be monitored and properly counseled to patients.

Finally, a commonly cited reason for antihypertensive nonadherence in previous studies is cost-related [150]. Providers should be careful to prescribe generic medications when available, as well as prescribing less-expensive antihypertensive medications if found to be therapeutically equivalent to more-expensive antihypertensive medications. In short, providers should pay keen attention to patient socioeconomic status and insurance coverage to recommend antihypertensives treatments in a financially sensitive manner. Similarly, suboptimal health literacy is repeatedly an area of concern regarding antihypertensive treatment nonadherence [151–153]. Given that hypertension is largely an asymptomatic disease, many patients may not understand the importance of strictly adhering to a prescribed antihypertensive treatment regimen. Thus, providers must carefully counsel older patients with hypertension on the importance of ongoing therapy, especially in those that are asymptomatic and without cardiovascular or metabolic comorbidities.

## 7. Future Directions

### 7.1. Research Priorities

Given the criticisms concerning generalizability common to each major RCT described in this review, the most pressing area of future work involves the inclusion of multimorbid and institutionalized patients with frailty in RCTs evaluating BP management in older adults. Such inclusion would directly address the lack of clarity and consensus in managing BP in the older population. Though ideal, including such individuals in RCTs is difficult, likely due to the stringent follow-up requirements RCT-enrolled patients must meet. However, recent developments have identified potential solutions to these issues. In 2021, the Health Care Systems Research Network–Older Americans Independence AGING Initiative sponsored a Stakeholder Advisory Panel intending to improve clinical research enterprise in nursing homes [154]. Solutions identified by the panel centered around establishing a nursing home clinical trials network to increase participation of institutionalized older patients in RCTs. Such involvement would help bridge the gap in understanding BP management in the older population with frailty.

Furthermore, given the importance of patient preference and shared decision-making that increases with age, as well as the increasing heterogeneity of patients among older age groups, better methods are needed to determine ideal BP targets at the individual level. A recent study conducted by Ascher et al. [155] in 2024 on individual-level SPRINT data simulated each patient’s preferences regarding the relative importance of cardiovascular events, cognitive impairment, death, and serious adverse events related to BP reduction. The investigators then compared net benefits as a weighted sum of risk differences across outcomes above and below each patient’s median adverse event risk [155]. They ultimately found that the majority of SPRINT participants favored intensive lowering of BP relative to standard treatment, yet the degree of net benefit varied considerably [155]. Thus, the investigators demonstrated a framework incorporating individual-level risks and preferences into BP control specific to SPRINT [155]. The limitations of the Ascher et al. study [155] include the omission of other factors important to decision-making, such as financial effects of treatment or complications. Therefore, prospective studies should expand on this work by investigating how the application of personalized BP targets may improve BP control in older adults at the individual level.

Finally, the questions of optimal BP levels and the permissibility of high BP in older patients with frailty or sarcopenia remain unanswered. There is likely a high degree of personalization required for each patient concerning optimal BP levels or allowance for more liberal BP goals. Regardless, future work should aim to identify subgroups of older patients with hypertension in which BP reduction confers more harm than benefit, especially since the available evidence thus far suggests it is never too late to treat hypertension. Furthermore, studies should employ advanced analytical tools such as machine learning and artificial intelligence to derive optimal values for various subgroups of the older population, including the multimorbid and institutionalized with frailty. The development of additional registries with proper follow-up concerning this patient population may also help increase the number of observational studies that may be conducted to address these questions [156].

### 7.2. Emerging Technologies

Several emerging technologies may further aid in managing BP in older adults. Firstly, digital technologies such as wearables and mobile applications have been demonstrated to improve the management of hypertension in a cost-effective manner [157,158]. Such technologies provide constant ambulatory and out-of-office monitoring of BPs, and providers may then remotely review all relevant data to inform subsequent decision-making [159]. The benefits of wearable technologies monitoring BP in older adults are even more important when considering the prevalence of frailty and institutionalization in this population limiting consistent access to in-person care [160]. Artificial intelligence developments may also provide benefits for risk stratification, personalized management, and prognostication of older patients with hypertension [161]. However, models utilizing machine learning or artificial intelligence principles are only as good as the data they are trained upon. This again points to the need for more registries reflecting older adults with hypertension that collect multi-omic (e.g., genomic, proteomic, and metabolomic) data, in addition to clinical, demographic, socioeconomic, behavioral, and environmental characteristics of these patients [161].

## 8. Conclusions

Hypertension in older adults is a complex, multifactorial process that may be complicated by age-related processes such as frailty and sarcopenia. Though there is prior evidence in observational studies cautioning against achieving low BPs in the older population with frailty, RCT-derived data and post hoc analyses support the role of intensive BP control in these patients, with potential benefits outweighing the risk of harms. Nevertheless, managing hypertension in older adults with frailty or sarcopenia requires careful evaluation of functional status and the ability to carry out ADLs. Providers must also thoughtfully consider patient and family preferences, life expectancy, and medication side effects in this fragile population. Future research evaluating BP management must address the limited inclusion of multimorbid and institutionalized individuals with frailty in previous trials to provide further clarity regarding BP management in older adults, as well as evaluate BP management at the individual level and identify subgroups that may benefit from higher BP levels.

## Figures and Tables

**Table 1.** ACC/AHA and ESC/ESH guidelines for classification of BP and treatment recommendations with qualifications in the older adult population.

Guideline Body	Stage	BP Definition *	BP Reduction Recommendations	BP Target	Qualifications for Older Adults
ACC/AHA [70]	Normal	SBP: <120 mmHg, DBP: <80 mmHg	Lifestyle optimization and yearly reassessment	SBP: <130 mmHg, DBP: <80 mmHg	N/a
	Elevated	SBP: 120–129 mmHg, DBP: <80 mmHg	Nonpharmacological therapy ** and reassessment in 3–6 months		
	Stage 1 hypertension	SBP: 130–139 mmHg, DBP: 80–89 mmHg	No clinical ASCVD and estimated 10-year risk < 10%: refer to elevated BP recommendations, clinical ASCVD or estimated 10-year risk ≥ 10%: nonpharmacological therapy **, BP-lowering medications, and reassessment in 1 month		Noninstitutionalized ambulatory community-dwelling adults ≥ 65 years of age: SBP goal of <130 mmHg Older adults (≥65 years of age) with comorbidities and limited life expectancy: reliance upon patient preferences, clinical judgment, multidisciplinary approach, and risk/benefit analysis
	Stage 2 hypertension	SBP: ≥140 mmHg, DBP: ≥90 mmHg	Nonpharmacological therapy **, BP-lowering medications, and reassessment in 1 month		
ESC/ESH [71]	Non-elevated	SBP: <120 mmHg, DBP: <70 mmHg	Lifestyle measures *** and opportunistic BP screening	SBP: 120–129 mmHg, DBP: 70–79 mmHg	N/a
	Elevated	SBP: 120–139 mmHg, DBP: 70–89 mmHg	Lifestyle measures ***, drug treatment, and monitoring of BP and medication tolerance		Older adults aged < 85 years without evidence of moderate to severe frailty: no changes in recommendations Older adults (≥85 years of age), pre-treatment symptomatic orthostatic hypotension, clinically significant moderate-to-severe frailty, or limited life expectancy (<3 years): follow outlined recommendations based on BP classification if BP ≥ 140/90 mmHg
	Hypertension	SBP: ≥140 mmHg, DBP: ≥90 mmHg			

BP: blood pressure. SBP: systolic BP. DBP: diastolic BP. mmHg: millimeters of mercury. ACC: American College of Cardiology. AHA: American Heart Association. ESC: European Society of Cardiology. ESH: European Society of Hypertension.

* Based on in-office BP measurements outlined by guideline bodies.

** Nonpharmacological interventions outlined by ACC/AHA [70] include weight loss, heart-healthy diet, sodium reduction, potassium supplementation, increased physical activity, and reduction of alcohol consumption.

*** Lifestyle measures outlined by ESC/ESH [71] include aerobic exercise training, increasing daily physical activity, avoiding sedentary lifestyle, isometric resistance training, potassium supplementation, optimizing weight management and diet, reducing sodium chloride intake, reducing alcohol intake, and smoking cessation.

**Table 2.** Reconciliation of clinical frailty and antihypertensive treatment recommendations in older adults. Adapted with permission from Benetos et al. [119].

Clinical Frailty Scale [73,74]	Benetos et al. [119] Classification	Benetos et al. [119] Treatment Recommendations
1—Very fit	Group 1—Preserved function	- Adhere to established ACC/AHA [70] or ESC/ESH [71] guidelines; - Start with monotherapy; - Titrate slowly.
2—Well		
3—Managing well		
4—Living with very mild frailty	Group 2—Loss of function/preserved activities of daily living (ADLs)	- Comprehensive geriatric assessment (CGA); - Adopt recommendations of group 1 or group 3 based on functional status.
5—Living with mild frailty		
6—Living with moderate frailty	Group 3—Loss of function and altered activities of daily living (ADLs)	- Adopt cautious approach; - Start with monotherapy; - Conservative goal: SBP < 150 mmHg; - Avoid >3 antihypertensives.
7—Living with severe frailty		
8—Living with very severe frailty		
9—Terminally ill		
